# Glyceraldehyde-3-phosphate dehydrogenase subunits A and B are essential to maintain photosynthetic efficiency

**DOI:** 10.1093/plphys/kiad256

**Published:** 2023-04-26

**Authors:** Andrew J Simkin, Mohammed Alqurashi, Patricia E Lopez-Calcagno, Lauren R Headland, Christine A Raines

**Affiliations:** School of Biosciences, University of Kent, Canterbury CT2 7NJ, UK; Department of Biological Sciences, University of Essex, Colchester CO4 3SQ, UK; Department of Biological Sciences, University of Essex, Colchester CO4 3SQ, UK; Department of Biological Sciences, University of Essex, Colchester CO4 3SQ, UK; School of Natural and Environmental Sciences, Newcastle University, Newcastle upon Tyne NE1 7RU, UK; Department of Biological Sciences, University of Essex, Colchester CO4 3SQ, UK; School of Molecular Biosciences, University of Glasgow, Glasgow G12 8QQ, UK; Department of Biological Sciences, University of Essex, Colchester CO4 3SQ, UK

## Abstract

In plants, glyceraldehyde-3-phosphate dehydrogenase (GAPDH; EC 1.2.1.12) reversibly converts 1,3-bisphosphoglycerate to glyceraldehyde-3-phosphate coupled with the reduction of NADPH to NADP^+^. The GAPDH enzyme that functions in the Calvin–Benson cycle is assembled either from 4 glyceraldehyde-3-phosphate dehydrogenase A (GAPA) subunit proteins forming a homotetramer (A_4_) or from 2 GAPA and 2 glyceraldehyde-3-phosphate dehydrogenase B (GAPB) subunit proteins forming a heterotetramer (A_2_B_2_). The relative importance of these 2 forms of GAPDH in determining the rate of photosynthesis is unknown. To address this question, we measured the photosynthetic rates of *Arabidopsis* (*Arabidopsis thaliana*) plants containing reduced amounts of the GAPDH A and B subunits individually and jointly, using T-DNA insertion lines of GAPA and GAPB and transgenic GAPA and GAPB plants with reduced levels of these proteins. Here, we show that decreasing the levels of either the A or B subunits decreased the maximum efficiency of CO_2_ fixation, plant growth, and final biomass. Finally, these data showed that the reduction in GAPA protein to 9% wild-type levels resulted in a 73% decrease in carbon assimilation rates. In contrast, eliminating GAPB protein resulted in a 40% reduction in assimilation rates. This work demonstrates that the GAPA homotetramer can compensate for the loss of GAPB, whereas GAPB alone cannot compensate fully for the loss of the GAPA subunit.

## Introduction

In recent years, there has been a focus to develop strategies to increase crop yields in order to feed the growing world population against the backdrop of climate change (IPCC 2014; Pereira 2017; IPCC 2019; NASA 2020). The photosynthetic capacity of a crop over the season determines the rate of growth and hence yield potential. A number of reports have now been published demonstrating that under glasshouse and field conditions, improvements in photosynthesis, including the Calvin–Benson-Bassham cycle (CBC), can improve the productivity and yield of the plant (Driever et al. 2017; Kubis and Bar-Even 2019; Simkin 2019; Simkin et al. 2019; Burgess et al. 2022; De Souza et al. 2022; Raines et al. 2022). In the CBC, glyceraldehyde-3-phosphate dehydrogenase (GAPDH) catalyzes the conversion of 1,3-bisphosphoglycerate to the triose phosphate, glyceraldehyde 3-phosphate (GAP) (Cséke and Buchanan 1986). Previous work has shown that the antisense suppression of the GAPDH gene had no effect on the rate of CO_2_ assimilation until GAPDH activity had decreased to 30% to 40% of WT levels (Price et al. 1995; Ruuska et al. 2000). However, more recently, a study showed that overexpression of GAPDH in rice (*Oryza sativa*) resulted in increased photosynthetic CO_2_ assimilation under elevated [CO_2_] conditions (Suzuki et al. 2021), raising the possibility that GAPDH could be a target for future manipulations to improve photosynthesis.

The CBC GAPDH is highly regulated and in plants is comprised of 2 distinct subunits, the glyceraldehyde-3-phosphate dehydrogenase A (GAPA) subunits and the glyceraldehyde-3-phosphate dehydrogenase B (GAPB) subunits that function as either as a homotetramer A_4_ or heterotetramer A_2_B_2_ (Cerff 1979; Iadarola et al. 1983; Howard et al. 2011). The primary structures of these 2 subunits show considerable similarity and are produced from separate nuclear genes (*GapA1*, *GapA2*, and *GapB*) (Cerff 1995). The GAPA subunits share 92.6% identity, and the major difference in the primary sequence between the GAPA and GAPB subunits is a C-terminal extension (CTE) on the GAPB, with substantial similarity to the C-terminus of chloroplast protein of 12 kDa (CP12) (Baalmann et al. 1996; Pohlmeyer et al. 1996). This CTE contains cysteine residues which have been shown to confer the thioredoxin-mediated redox regulatory capacity onto the GAPDH A_2_B_2_ complex (Baalmann et al. 1996; Scheibe et al. 1996; Sparla et al. 2002; Marri et al. 2005; Fermani et al. 2007).

In the chloroplast of vascular plants, the predominant active form of GAPDH is believed to be the A_2_B_2_; however, leaves of many species contain other, less abundant forms of GAPDH including the A_4_ form and the 2(A_2_B_2_) and 4(A_2_B_2_) multimers involved in deactivation of the enzyme (Wolosiuk and Buchanan 1976; Scagliarini et al. 1998; Sparla et al. 2005; Fermani et al. 2007). The homotetramer A_4_ form of GAPDH has been termed “nonregulatory” (GAPDH_N_), firstly because of the absence of the CTE identified in GAPB and secondly because it fails to aggregate into larger oligomers and the A_2_B_2_ regulatory form (GAPDH_R_) (Scagliarini et al. 1998). In the absence of the CTE, GAPDH_N_ is thought to be regulated by the formation of the CP12/GAPDH/PRK (phosphoribulokinase) complex (Trost et al. 2006; Lopez-Calcagno et al. 2014). Although the regulation of the 2 tetramers of GAPDH is different, results of Scagliarini et al. (1998) showed that the kinetic properties of GAPDH_N_ are similar to GAPDH_R_. The activity data for the GAPDH A_4_ and the A_2_B_2_ showed that both of these isoforms have similar kinetic parameters, with a *V*_max_ (NADPH) of 130 and 114 *µ*mol min^−1^ mg^−1^, respectively, and a Km (BPGA) of 2.0 and 2.3 *µ*M, respectively. Based on these data, it was proposed that that the B-subunits are mostly responsible for the regulation of the enzyme (Sparla et al. 2005) and the A-subunits for catalytic activity (Scagliarini et al. 1998). Over and above the multimers of the A_2_B_2_ complex, a further level of redox regulation of GAPDH activity occurs through the formation of a high-molecular weight complex which includes the CBC enzyme PRK and the small regulatory protein CP12 (Howard et al. 2008; Carmo-Silva et al. 2011; Lopez-Calcagno et al. 2014; Lopez-Calcagno et al. 2017).

The A_4_ homotetramer has been shown in spinach (*Spinacia oleracea*) chloroplast preparations to constitute 15% to 20% of the total GAPDH activity (Scagliarini et al. 1998). Howard et al. (2011) examined stromal extracts from dark-adapted leaves of species from Leguminosae (pea [*Pisum sativum* ‘Onwards’], *Medicago* [*Medicago truncatula* ‘Jemalong’], broad bean [*Vicia faba* ‘The Sutton’], French bean [*Phaseolus vulgaris* ‘Vilbel’]), Solanaceae (potato [*Solanum tuberosum* ‘Desiree’], tomato [*Solanum lycopersicon* ‘Gardener's Delight’], and tobacco [*Nicotiana tabacum* ‘Samson’]), Amaranthaceae (spinach), and the Brassicaceae (*Arabidopsis*). This study revealed that the relative amounts of the A_2_B_2_ and the A_4_ complexes vary among species. Whereas all species were found to accumulate the A_2_B_2_ heterotetramer, in contrast, in some plant species, the A_4_ tetramer was not detected (Howard et al. 2011). This raises the question of the role of the A_4_ form for the activity of GAPDH in the CB cycle. To date, the relative importance of the A_4_ versus the A_2_B_2_ form of plastid GAPDH, in determining the rate of CO_2_ assimilation, has not been elucidated. In this manuscript, to explore this question, we have used insertion mutants in both the *GapB* and *GapA-1* genes together with transgenic lines where the relative amounts of the GAPA and GAPB proteins have been decreased individually.

## Results

### Identification and analysis of *Arabidopsis* lines with reductions in GAPDH A and B transcript and protein levels

**Figure 1.**
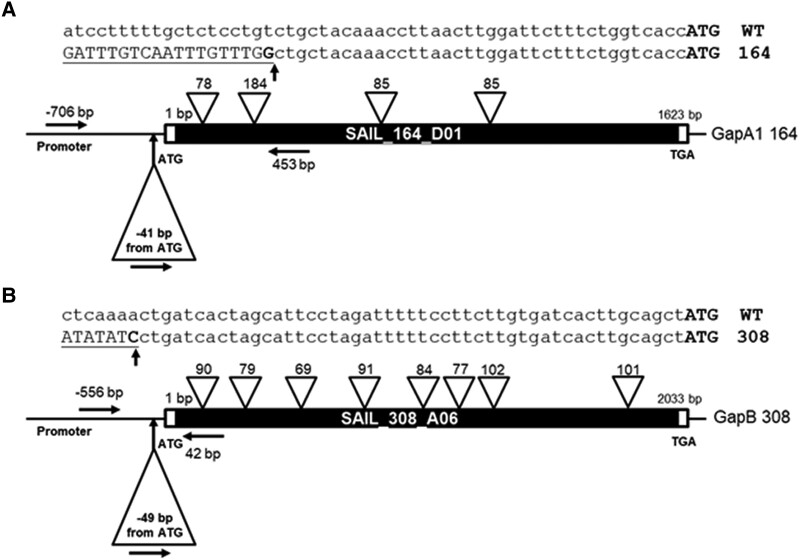
Molecular analysis of homozygous GAPDH T-DNA insertion mutants. Structure of the 2 GAPDH genes and the location of T-DNA insertions in the **A)***gapa-1* (At3g26650; SAIL_164_D01) and **B)***gapb* (At1g42970; SAIL_308_A06) mutants. Protein-coding exons are represented by the solid bar, and intron locations are displayed as inverted white triangles above the coding sequence. The location of genomic PCR screening primers is shown by black arrows on each gene model. T-DNA insertion sites are indicated by triangles below the sequence, and the precise position is given as the number of base pairs from the ATG. ATG, translation initiation codon; TGA, translation termination codon. Bolded G **A)** and C **B)** indicate the point of sequence insertion into the promoter region of the SAIL_164_D01 and SAIL_308_A06 mutants.

**Figure 2.**
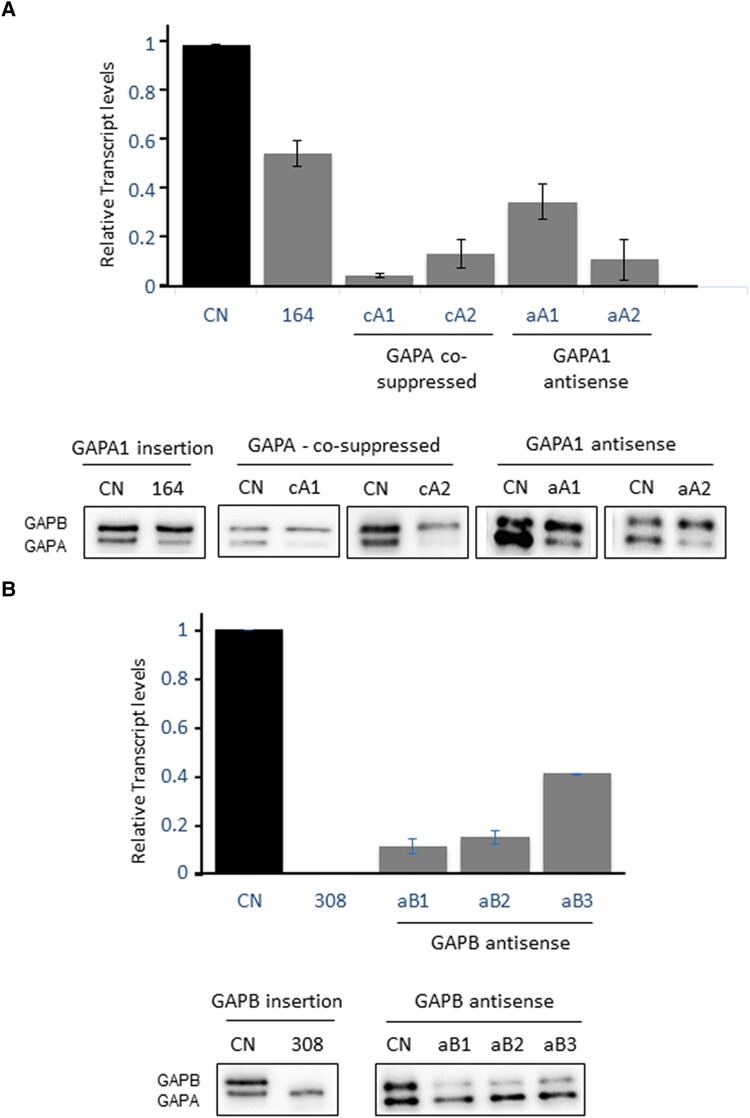
RT-qPCR and immunoblot analysis of leaf proteins of wild-type and experimental GAPDH plants. **A)** Transcript and protein levels in *gapa-1* (At3g26650; SAIL_164_D01), GAPA cosuppressed lines (cA), GAPA antisense lines (aA), and control (CN). **B)** Transcript and protein levels in *gapb* (At1g42970; SAIL_308_A06) and GAPB antisense lines (aB). Protein (6 *µ*g) extracts from leaf disks taken from 2 leaves per plant and separated on a 12% acrylamide gel, transferred to membranes, and probed with antibodies to GAPDH which recognizes both GAPA and GAPB subunits. Error bars represent Se of 3 plants per line.

We identified T-DNA insertion mutants for *gapa-1* (SAIL_164_D01) and *gapb* (SAIL_308_A06) from The Arabidopsis Information Resource (TAIR) database (http://www.arabidopsis.org/). All T-DNA insertion sites were confirmed using PCR analysis of genomic DNA followed by sequencing of the T-DNA/gene junctions. The positions of the T-DNA inserts are presented in Fig. 1. Homozygous plants (identified by PCR) were used to assess the effect of each T-DNA insertion on the expression of the GAPDH transcripts. RT-qPCR analysis confirmed that the transcript abundance encoding the GAPA subunit in the *gapa-1* mutant was reduced by approximately 45%, the remaining transcript being produced by the *gapa-2* gene (Fig. 2A). The transcript for the GAPB subunit in the *gapb* mutant was not detected in the T-DNA insertion line, evidencing the *gapb* mutant as a true knockout (KO) (Fig. 2B). In order to obtain additional independent mutant lines, GAPA and GAPB expression levels were downregulated using antisense constructs (Figs. 2, A and B, and S1). Additionally, 2 GAPA cosuppressed lines were identified from plants transformed with a GAPA overexpression construct using the *Arabidopsis* sequence in the sense orientation (Supplemental Fig. S1C). In *GapA* cosuppressed transformants, we identified 2 lines with 5% (cA1) and 11% (cA2) of total *GapA* (*GapA-1* and *GapA-2*) transcript levels and antisense lines with 35% (aA1) and 11% (aA2) of the *GapA* transcript (Fig. 2A). In antisense *GapB* transformants, we identified 3 lines with 11% (aB1), 15% (aB2), and 41% (aB2) levels of the *GapB* transcript (Fig. 2B). Western blot analysis of these mutant lines was used to determine changes in GAPDH protein levels, which showed a reduction in bands at 37.6 kDa representing GAPA and 47.7 kDa (Fig. 2, A and B). In the GAPA1 insertion line and the GAPA1 antisense lines, the level of the GAPA protein was reduced to 51% to 43% of the CN plants, and in the GAPA cosupressed lines, only 9% of the GAPA protein was detected (Table 1). In the GAPB insertion line, no band was detected indicating the absence of the B subunit in this mutant (Fig. 2A). The level of protein in the GAPB antisense lines was between 15% and 40% of the CN plants (Fig. 2B and Table 1).

**Table 1.** Molecular and physiological analysis of wild type (WT) and GAPDH lines

Line	Relative % *GapA* transcript and protein	Relative % *GapB* transcript and protein	*F*_q_ʹ/*F*_m_ʹ 600 *µ*mol m^−2^ s^−1^	*J*_max_	*V*c_max_	*A*_max_
CN	WT	WT	0.478 ± 0.014	145.5 ± 15.75	55.1 ± 5.2	28.3 ± 1.15
164	**55.2 15.5** ***51.6*** ± ***1.9***	WT	0.479 ± 0.004	**89.0 ± 11.12**	**40.2 ± 5.62**	**16.2 ± 2.71**
cA	**9.4 ± 4.4** ***9.1* ± *3.7***	WT	**0.420 ± 0.014**	**51.9 ± 4.41**	**37.6 ± 3.19**	**7.67 ± 0.91**
aA	**23.3 ± 11.8** ***43.6* ± *5.1***	WT	0.449 ± 0.006	**119.7 ± 7.90**	**40.2 ± 2.67**	**23.4 ± 1.38**
308	WT	**ND**	0.469 ± 0.003	**101.8 ± 8.91**	56.4 ± 1.12	**18.8 ± 2.27**
aB	WT	**22.3 ± 9.34** ***26.1* ± *13.8***	0.453 ± 0.004	**110.5 ± 17.12**	52.3 ± 5.92	**18.2 ± 2.34**
164/ 308	**55.2 ± 15.5** ***51.6* ± *1.9***	**ND**	**0.450 ± 0.012**	**101.1 ± 4.44**	**38.4 ± 3.49**	**20.1 ± 0.54**

**Figure 3.**
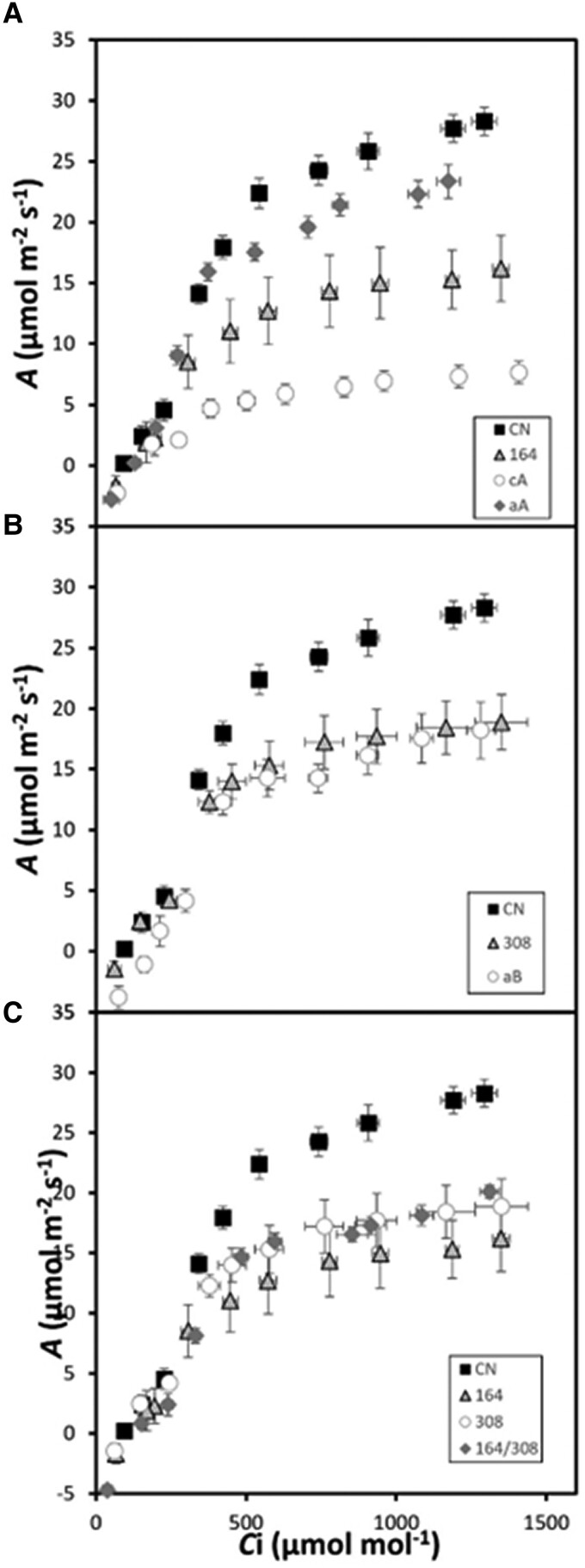
Photosynthetic carbon fixation rate determined as a function of increasing CO_2_ concentrations (*A*/*C*_i_) at saturating light levels (1,000 *µ*mol m^−2^ s^−1^). **A)** Controls (CN) compared to the *gapa-1* insertion line (164) and GAPA cosuppressed (cA) and GAPA antisense (aA) lines. **B)** CN compared to *gapb* insertion line (308) and GAPB antisense (aB) line lines, and **C)** photosynthetic carbon fixation of CN compared to single insertion mutants *gapa-1* (164) and *gapb* (308) and the double-mutant *gapa-1/gapb* (164/308). Extrapolated data are in Table 1. Error bars represent Se of 6 plants per line.

The quantum efficiency of photosystem II (*F*_q_'/*F*_m_'), the maximum electron transport rate (*J*_max_), the maximum rate of carboxylation by Rubisco (*V*c_max_), and maximum assimilation (*A*_max_) in control and GAPDH lines in relation to reported protein levels. Plants were grown in short days at 130 *µ*mol m^−2^ s^−1^ light intensity, 8-h light/16-h dark cycle. Values represent 4 to 6 plant independent lines (6 to 8 plants) for the group. *A*_max_, *V*c_max_, and *J*_max_ derived from *A*/*C*_i_ response curves shown in Fig. 3 using the equations published by von Caemmerer and Farquhar (1981) using the spreadsheet provided by Sharkey (2016). Statistical differences are shown in boldface (*P* < 0.05). Se are shown. Protein quantities are shown in italics. ND, not detected; WT, plants containing wild-type levels of the transcript and protein subunit; CN, control; 164, *gapa-1* insertion; 308, *gapb* insertion; cA, cosupressed GapA; aA, antisense GapA; aB, antisense GapB.

### Chlorophyll fluorescence imaging reveals that PSII efficiency is maintained in plants showing a significant reduction in GAPA protein levels

In order to explore the impact of a decrease in the subunits of the GAPDH enzyme on the photosynthetic capacity, the quantum efficiency of PSII (*F*_q_′/*F*_m_′), chlorophyll *a* fluorescence was analyzed (Baker 2008; Murchie and Lawson 2013). No significant decrease in *F*_q_′/*F*_m_′ was observed in the *gapa-1* insertion line 164 which had a 45% reduction in *GapA* transcript levels and 46% reduction in GAPA protein or in the *gapb* insertion line 308 with no detectable level of the GAPB protein (Table 1). The GapA antisense lines, containing 46% of WT protein levels, maintained equivalent PSII photosynthetic efficiency to controls. GapB antisense lines also showed no significant differences in *F*_q_′/*F*_m_′ consistent with the observed result in the *gapb* insertion line 308, suggesting that in the absence of GAPB, the presence of the GAPDH A subunit is sufficient to maintain the photosynthetic capacity of these plants.

A small decrease of 12% in *F*_q_′/*F*_m_′ was found in the *GapA* cosuppressed lines that had the lowest level of GAPA protein (Table 1). To investigate further the impact of a combined reduction of both the GAPA and GAPB protein levels, double mutants *gapa-1/gapb* (164/308) were generated. Homozygous plants with insertions in both *gapa-1* (164) and *gapb* (308) were grown as described above. Interestingly, double mutants of *gapa-1*/*gapb* (164/308) showed a significant reduction in *F*_q_′/*F*_m_′ suggesting that a GAPA protein level of 52% is insufficient to maintain photosynthetic efficiency in the absence of GAPB.

### Photosynthetic CO_2_ assimilation and electron transport rates are reduced in lines with a reduced GAPDH protein level

To assess the impact on photosynthesis of changes in the levels of GAPDH protein, CO_2_ assimilation rates were determined as a function of internal CO_2_ concentration (*C*_i_) (*A*/*C*_i_′ curve). Plants were grown in environmentally controlled chambers under short-day conditions as described in materials and methods. The gas exchange measurements were made on mature leaves on plants 6 wk after germination. *A*/*C*_i_ curves were determined for the *gapa-1* insertion line (164), *gapb* insertion line (308), cosuppressed line of *GapA* (cA), *GapA* antisense line (aA), *GapB* antisense line (aB), and *gapa-1/gapb* crossed line (164/308) compared to the control (CN) plants (Fig. 3). From these *A*/*C*_i_ curves, the maximum rate of CO_2_ assimilation (*A*_max_) in all mutant lines tested was shown to be significantly lower than that for the CN plants. The plants with the lowest levels of the GAPA protein had the greatest decrease in assimilation rate (Fig. 2A), with maximum assimilation rates attained in these plants being approximately 27% of that observed in the CN (Fig. 3A and Table 1). Furthermore, in the *gapa-1* mutant (164), an approximately 50% reduction in GAPA protein levels resulted in a 40% reduction in maximum assimilation (Fig. 3A).

Plants with no detectable level of GAPB protein (Fig. 2B) had a 30% decrease in assimilation rates compared to the 73% reduction observed in a line with 9% GAPA proteins (cA) (Fig. 3B). Finally, in line 164/308, representing the double-mutant *gapa-1*/*gapb*, containing no GAPB protein and only 51% of the levels of GAPA protein, the assimilation rates are similar to the single *gapa-1* (164) and *gapb* (308) insertion mutants. This result suggests that the double mutant shows no cumulative impact on assimilation rates under these conditions as long as 51% GAPA protein remains (Fig. 3C).

Further analysis of the *A*/*C*_i_ curves using the equations published by von Caemmerer and Farquhar (1981) illustrated that the maximum rate of carboxylation by Rubisco (*V*c_max_) and maximum electron transport rate (*J*_max_) were reduced in some lines (Sharkey et al. 2007; Sharkey 2016) (Table 1). The results for *V*c_max_ showed that lines with a reduction in GAPA displayed a significant decrease compared to CN. No significant difference in *V*c_max_ was observed in plants with a reduction in GAPB.

Furthermore, the results showed that the lines with reductions in either GAPA or GAPB had a lower rate of photosynthetic electron transport (*J*_max_), needed to sustain ribulose 1,5-bisphosphate (RuBP) regeneration, when compared to control plants (Table 1). As previously noted, the maximum rate of CO_2_ assimilation (*A*_max_) was significantly lower in all lines compared to CN; however, *A*_max_ was significantly lower in cA, where *GapA* transcript and GAPA protein levels were at the lowest levels. No significant differences in *A*_max_ were observed between the single mutants *gapa-1* (164) and *gapb* (308) compared to the double-mutant *gapa-1/gapb* (164/308).

### Growth and vegetative biomass are reduced in both GAPA- and GAPB-reduced lines

**Figure 4.**
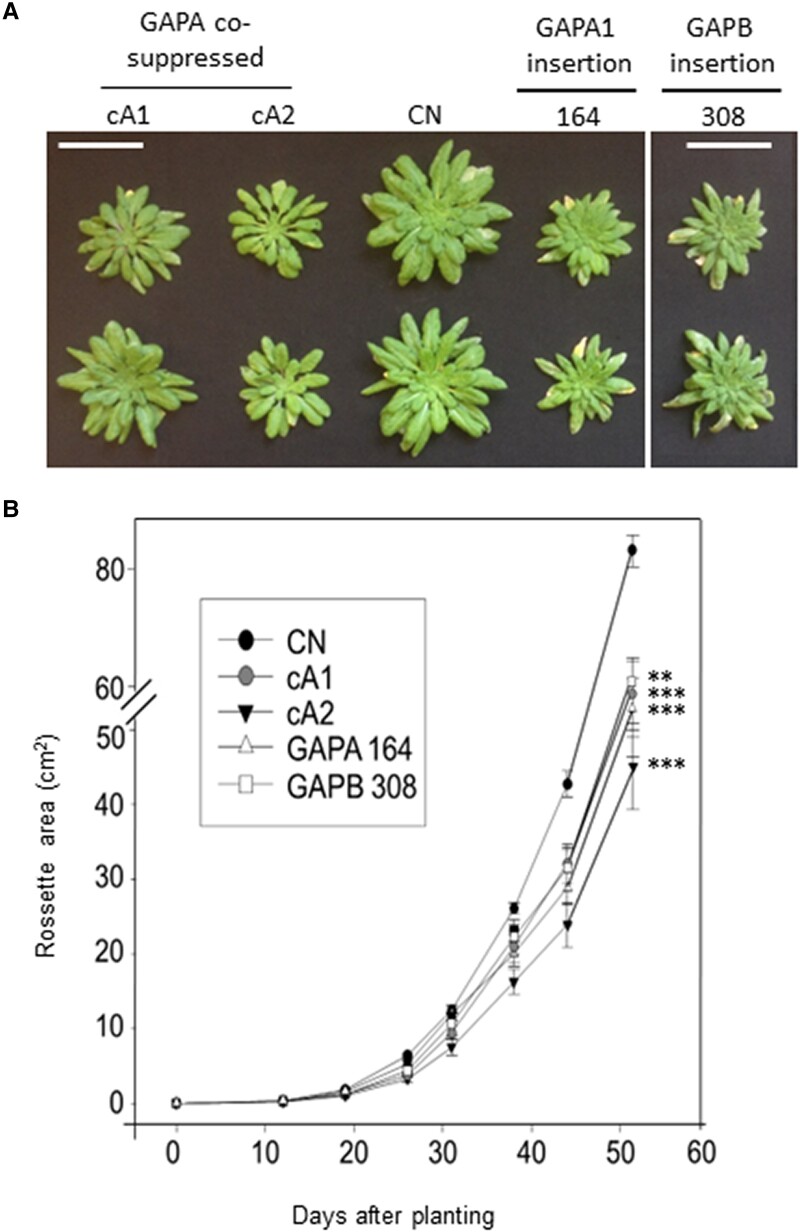
Growth analysis of control and experimental lines grown. **A)** Plants were grown at 130 *µ*mol m^−2^ s^−1^ light intensity in short days (8 h/16 h/d) for 52 d. White bar represents a size of 6 cm. **B)** Plant growth rate evaluated over the 1st 52 d. Lines cosuppressing GAPA (cA), controls (CN), *gapa-1* insertion mutant (164), and *gapb* insertion mutant (308) are represented. Results are representative of 9 to 12 plants per line (CN plants include azygous lines segregated from primary transformants). Significant differences **P < 0.05, and ***P < 0.01 are indicated. Unless indicated, results are presented as a percentage of CN (CN = 100%). Error bars represent Se.

Growth analysis of GapA cosuppressed and insertion lines was carried out on homozygous plants grown in growth chambers at 22 °C under a short-day length (130 *µ*mol m^−2^ s^−1^ in an 8-h/16-h light/dark cycle) and relative humidity (RD) 50%. The growth rate of these plants was determined using image analysis of the total leaf area over a period of 52 d from planting. Observations of the growth rates of *GapA* cosuppressed lines (cA), CN Columbia (Col-0), and the *gapa-1* and *gapb* insertion lines (164 and 308) showed a statistically significant reduction in all growth parameters (Figs. 4 and Supplemental Fig. S2). The cosuppressed and insertion lines were shown to have a statistically significantly slower growth rate when compared to the CN plants at 40 d post planting (Fig. 4, A and B). By 52 d post planting, this growth trend continued (Fig. 4B) and the final leaf area was reduced compared to controls (Fig. 4B).

**Figure 5.**
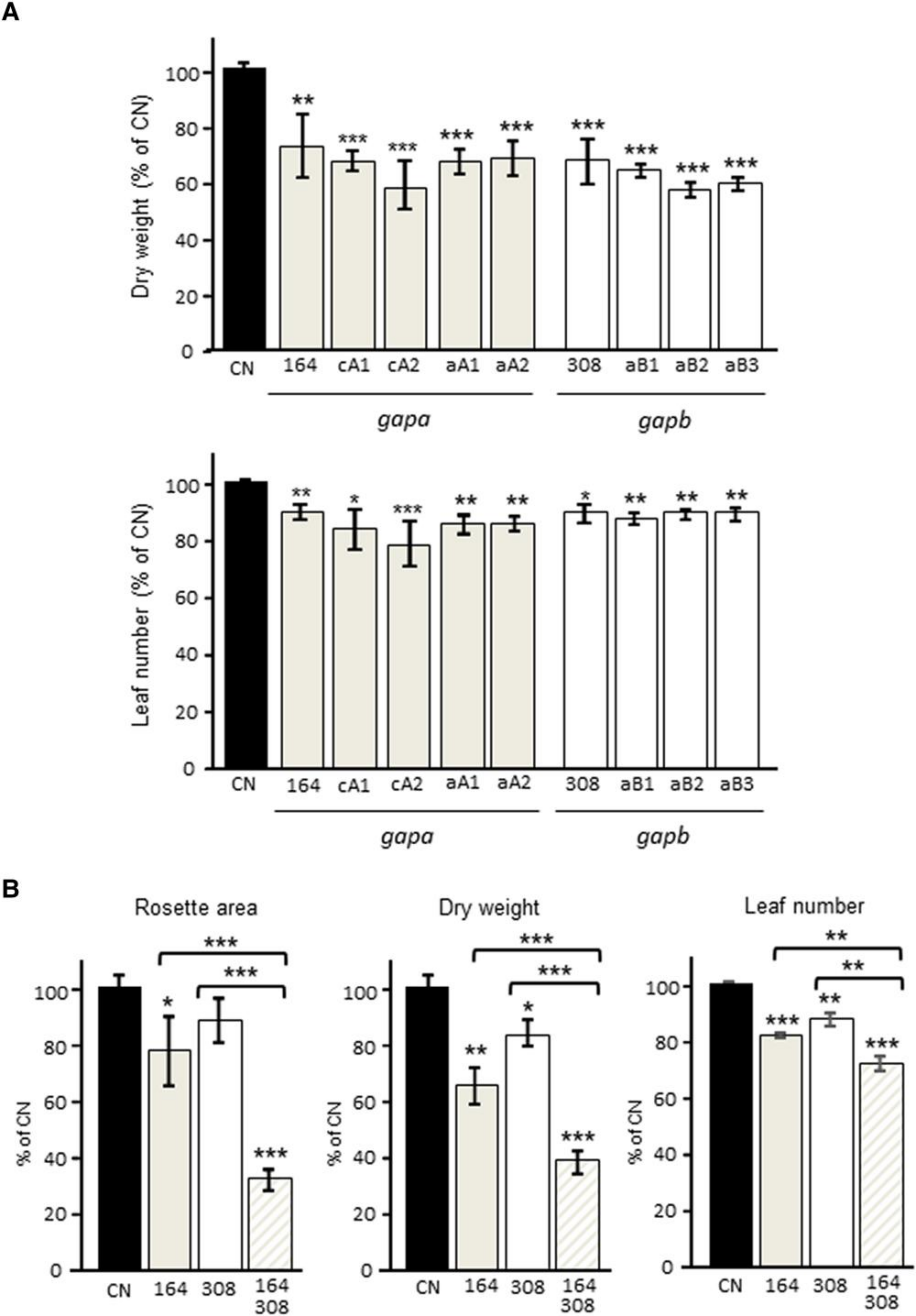
Growth analysis of control and experimental lines grown in low light. **A)***gapa-1* (164) and *gapb* (308) insertion mutants and GAPA cosuppressed (cA), GAPA antisense (aA), and GAPB (aB) antisense lines were analyzed in parallel. Results are representative of 8 plants per line. **B)***gapa-1* (164) and *gapb* (308) insertion mutants and the double-mutant *gapa-1/gapb* (164/308) crosses were evaluated. Results are representative of 9 to 12 plants per line. Plants were grown at 130 *µ*mol m^−2^ s^−1^ light intensity in short days for 46 d (CN plants include azygous lines segregated from primary transformants). Data were statistically analyzed using 2-way ANOVA. Significant differences *P < 0.10, **P < 0.05, and ***P < 0.01 are indicated. Results are presented as a percentage of CN (CN = 100%). Error bars represent Se.

A growth analysis of the *gapa-1* insertion mutant (164), the *GapA* cosuppressed (cA), and the *GapA* antisense lines (aA) showed a significant reduction in dry weight and leaf number (Fig. 5A) compared to the CN. Significant reductions in the leaf number and final biomass were seen in the *gapb* insertion mutant (308), and *GapB* antisense lines were also observed when compared to CN (Fig. 5B). A comparative analysis of the single insertion mutants *gapa-1* and *gapb* with the double mutants *gapa-1/gapb* showed that reduction in both the A and B subunits resulted in a greater decrease in leaf area, biomass, and leaf number after 46 d of growth (Fig. 5B), even in the absence of a larger decrease in assimilation rates observed in Fig. 3C (see Table 1).

## Discussion

### A reduction in GAPA protein levels inhibits CO_2_ assimilation and reduces biomass yield

Previous research showed that a 60% to 70% reduction in GAPDH activity was needed to affect growth and development in tobacco (*N. tabacum* cv. ‘W38’) antisense GAPDH lines and that no severe impact on photosynthesis was observed until levels were reduced to less than 35% of wild-type levels (Price et al. 1995). The results presented in this study clearly showed a slow growth phenotype in *A. thaliana* following reductions in GAPA protein levels by 50%. *GapA* cosuppressed lines, with more than a 90% reduction in *GapA* transcript levels and a barely detectable GAPA protein content, showed the most statistically significant impact on photosynthetic efficiency (−73%) even with GAPB being present at wild-type levels. The principal form of GAPDH in plant chloroplast has been proposed to be the heterotetrameric A_2_B_2_. In some plants, including spinach, an A_4_ homotetramer has also been detected representing up to 20% of the total GAPDH activity (Scagliarini et al. 1998). This A_4_ homotetramer was not detected in *Arabidopsis* by previous studies (Howard et al. 2011), providing evidence that under normal circumstances, the A_2_B_2_ tetramer is the principal active form in *Arabidopsis*. In this study, plants showing an absence of GAPB protein in the *gapb* mutant lines maintained photosynthesis rates at 66% of wild-type levels suggesting that under conditions where GAPB is limiting, or absent, the A_4_ form of GAPDH can maintain photosynthesis.

Importantly, the work here also allowed a comparative analysis between plants with different levels of the GAPA and GAPB subunits under the same environmental conditions. When the *gapa-1* (164) and *gapb* (308) mutant lines were crossed to form the double-mutant *gapa-1/gapb* (164/308), the combined effects resulted in a cumulative reduction in biomass (−60%), which was significantly greater than the reductions observed in the *gapa-1* (−35%) or *gapb* (−16%) mutants alone. Interestingly, the assimilation rates for the *gapa-1/gapb* double mutant showed no further reductions compared to *gapa-1* and *gapb* single mutants. This suggests that, even though GAPA levels are reduced, GAPA is able to maintain the assimilation rate even in the absence of GAPB and secondly, that the decrease in biomass observed in the double mutant may be due to impacts early in development leading to a cumulative effect on growth.

Recent reviews of the literature have shown that overexpressing of some CBC enzymes can lead to increases in photosynthesis and biomass and that a multitarget approach can result in cumulative yield gains in some plants (Simkin 2019; Simkin et al. 2019; Raines 2022). The co-overexpression of *GapA* and *GapB* in transgenic rice increased the GAPDH activity to more than 300% of the wild-type levels; under elevated [CO_2_], CO_2_ assimilation increased by approximately 10% demonstrating that the overproduction of the chloroplast GAPDH proteins is effective at improving photosynthesis at least under elevated [CO_2_]. However, under these conditions, no statistically significant differences in biomass were observed compared to wild-type plants, although a small increase in starch accumulation was observed. (Suzuki et al. 2021). In contrast, no statistically significant difference in CO_2_ assimilation was observed in ambient [CO_2_] (Suzuki et al. 2021). These results suggest that the manipulation of GAPDH activity may have more importance as atmospheric [CO_2_] increases due to the current climate change models where [CO_2_] increases from 416 ppm to 550 by 2050 and 700 ppm by 2100 (Le Quéré et al. 2009; IPCC 2019; NASA 2020). Furthermore, given that no increase in growth rate or final biomass was observed at ambient [CO_2_], increasing GAPDH may have more value in a multitarget approach, such as targeting additional CBC enzymes, photorespiratory elements, and photosynthetic electron transport in combination with GAPDH in the same plants.

## Conclusion

Our results have shown that both GAPA and GAPB are essential for normal growth and development in *Arabidopsis* plants and that the A_2_B_2_ form of the enzyme is required for maximum photosynthetic efficiency. The phenotypes described in this manuscript provide in vivo evidence of the relative importance of the individual subunits of the GAPDH complex on photosynthetic carbon assimilation. In this study, we also show that the suppression of GAPA to almost undetectable levels resulted in a 73% decrease in carbon assimilation compared to 34% reduction in photosynthesis in the absence of GAPB providing direct evidence of the importance of GAPA in maintaining photosynthetic capacity.

## Materials and methods

### Identification and analysis of T-DNA GAPDH mutants and production of double mutants

The *gapa-1* and *gapb* mutants in *Arabidopsis* (*Arabidopsis thaliana*) were identified in the Arabidopsis Information Resource (TAIR) database (*gapa-1*: SAIL_164_D01 and *gapb*: SAIL_308_A06). The mutant insertion sites were identified by PCR, and the location of each T-DNA insertion was determined by sequencing the PCR products spanning the junction site (Fig. 1). The *GapA-1* was amplified with forward primers GapA1 Fwd (5′gagagcatgtgacataacggg′3) and reverse primer GapA1 Rev (5′accttaagcttggcctcagtc′3) in conjunction with primer Sail_LB3 (5′tagcatctgaatttcataaccaatctcgatacac′3). The *GapB* was amplified with forward primers GapB Fwd (5′cgacgatgtctcctctcagc′3) and reverse primer GapB Rev (5′gaccgggattcttgagacg′3) in conjunction with primer Sail_LB3. Double mutants *gapa-1/gapb* (164/308) were obtained by crossing homozygous plants of *gapa-1* (SAIL_164_D01) and *gapb* (SAIL_308_A06) and segregating the double homozygous plants.

### Construct generation

#### GAPA and GAPB antisense constructs

A partial-length coding sequence of GAPA (*GapA-1*: At3g26650) and GAPB (*GapB*: At1g42970) subunits was amplified by RT-PCR using primers AtGAPAf (5′cacctatcgaaggaaccggagtgtt′3) and AtGAPAr (5′tcctgtagatgttggaacaatg′3) and AtGAPBf (5′caccttgatggtaagctcatcaaagtt′3) and AtGAPBr (5′ggtgtaggagtgtgtggttgt′3), respectively. The resulting amplified products were cloned into pENTR/D (Invitrogen, UK) to make pENTR-GAPA1: pENTR-*antiGAPA* and pENTR-*antiGAPB.* The cDNAs were introduced into the pGWB2 gateway vector (Nakagawa et al. 2007) AB289765 by recombination from the pENTR/D vector to make pGWB2-AntiGAPA and pGWB2-AntiGAPB (Supplemental Fig. S1). cDNAs are under transcriptional control of the 35s tobacco mosaic virus promoter, which directs constitutive high-level transcription of the transgene, and followed by the *nos* 3′ terminator.

#### GAPA-1 sense constructs

Destination vector pGWPTS1 was generated as described in Simkin et al. (2017a). The full-length coding sequencer of *GapA-1* was amplified using primers AtFwd (5′caccatggcttcggttactttctctgtcc′3) and AtRev (5′ttgatgaaatcacttccagttgttgg′3). The resulting amplified product was cloned into pENTR/D (Invitrogen, UK) to make pENTR-AtGAPA-1, and the sequence was verified and found to be identical. The full-length cDNA was introduced into destination vector pGWPTS1 by recombination from the pENTR/D vector to make pGWPTS1-AtGAPA-1 (PTS1-GAPA-1) (Supplemental Fig. S1). The transgene was under the control of the rbcS2B (1150 bp; At5g38420) promoter. In this instance, the expression of the cDNA was under transcriptional control of the Rubisco small subunit 2B (rbcS2B) promoter (At5g38420), which directs high-level photosynthetic tissue-specific transcription of the transgene and followed by the *nos* 3′ terminator.

### Generation of transgenic plants

The recombinant plasmid pGWB2-AntiGAPA, pGWB2-AntiGAPB, and pGWPTS1-GAPA-1 were introduced into wild-type *Arabidopsis* by floral dipping (Clough and Bent 1998) using *Agrobacterium tumefaciens* GV3101. Positive transformants were regenerated on MS medium containing kanamycin (50 mg L^−1^). Kanamycin-resistant primary transformants (T1 generation) with established root systems were transferred to soil and allowed to self-fertilize. Full details of pGWB2-AntiGAPA, pGWB2-AntiGAPB, and PTS1-GAPA-1 construct assembly can be seen in the Supplemental Fig. S1.

### Plant growth conditions

For the experimental study, T3 progeny seeds from selected lines were germinated on soil in controlled environment chambers at an irradiance of 130 *µ*mol photons m^−2^ s^−1^, 22 °C, relative humidity of 60%, in an 8 h/16 h square-wave photoperiod. Plants were sown randomly, and trays were rotated daily. Leaf areas were calculated using standard photography and ImageJ software (imagej.nih.gov/ij). Wild-type plants and null segregants (azygous) used in this study were evaluated independently. Once it was determined that no substantial differences were observed between these 2 groups, wild-type plants and null segregants were combined (null segregants from the transgenic lines verified by PCR for nonintegration of the transgene) and used as a combined “control” group (CN) (Supplemental Fig. S3). Four leaf disks (0.6 cm diameter) from 2 individual leaves, were taken and immediately plunged into liquid nitrogen and stored at −80 °C for determination of transcript levels by RT-qPCR and protein content by western blot.

### cDNA generation and Rt-qPCR

Total RNA was extracted from *Arabidopsis* leaf using the NucleoSpin RNA Plant Kit (Macherey-Nagel, Fisher Scientific, UK). cDNA was synthesized using 1 *µ*g total RNA in 20 *µ*L using the oligo-dT primer according to the protocol in the RevertAid Reverse Transcriptase kit (Fermentas, Life Sciences, UK). cDNA was diluted 1 in 4 to a final concentration of 12.5 ng *µ*L^−1^. For semiquantitative RT-PCR, 2 *µ*L of RT reaction mixture (100 ng of RNA) in a total volume of 25 *µ*L was used with DreamTaq DNA Polymerase (Thermo Fisher Scientific, UK) according to manufacturer's recommendations. For RT-qPCR, the SensiFAST SYBR No-ROX Kit was used according to the manufacturer's recommendations (Bioline Reagents Ltd., London, UK). GAPA-1 (At3g26650) and GAPA-2 (At1g12900) transcripts were amplified using primers GAPA-F (5′atggttatgggagatgatatgg′3) and GAPA-R (5′ttattggcaacaatgtcagcc′3) and GAPB-F (5′ttcaggtgctctgatgtctctacc′3) and GAPB-R (5′tagccactaggtgagccaaatccacc′3), respectively.

### Protein extraction and western blotting

Total protein was extracted in extraction buffer (50 mM 4-[2-hydroxyethyl]piperazine-1-ethanesulfonic acid [HEPES] pH 8.2, 5 mM MgCl2, 1 mM ethylenediaminetetraacetic acid tetrasodium salt [EDTA], glycerol 10% *v*/*v*, Triton X-100 0.1% *v*/*v*, 2 mM benzamidine, 2 mM aminocaproic acid, 0.5 mM phenylmethanesulfonyl fluoride [PMSF], and 10 mM DTT). Any insoluble material was removed by centrifugation at 14,000 g for 10 min (4 °C), and protein quantification was determined as previously described (Harrison et al. 1998; Simkin et al. 2017a). Samples were loaded on an equal protein basis, separated using 12% (*w*/*v*) SDS-PAGE, transferred to polyvinylidene difluoride membrane, and probed using antibodies raised against GAPDH (Pohlmeyer et al. 1996). Proteins were detected using horseradish peroxidase conjugated to the secondary antibody and ECL chemiluminescence detection reagent (Amersham, Buckinghamshire, UK).

### Chlorophyll fluorescence imaging screening in seedlings

Measurements were performed on 2-wk-old *Arabidopsis* seedlings that had been grown in a controlled environment chamber at 130 *µ*mol mol^−2^ s^−1^ photosynthetic photon flux density (PPFD) and ambient CO_2_.Chlorophyll fluorescence parameters were obtained using a chlorophyll fluorescence (CF) imaging system (Technologica, Colchester, UK; Barbagallo et al. 2003; von Caemmerer et al. 2004). The operating efficiency of photosystem 2 (PSII) photochemistry, *F*_q_′/*F*_m_′, was calculated from measurements of steady-state fluorescence in the light (*F*′) and maximum fluorescence in the light (*F*_m_′) since *F*_q_′/*F*_m_′ = (*F*_m_′−*F*′)/*F*_m_′. Images of *F*′ were taken when fluorescence was stable at 130 *µ*mol m^−2^ s^−1^ PPFD, while images of maximum fluorescence were obtained after a saturating at 600 ms pulse of 6,200 *µ*mol m^−2^ s^−1^ PPFD (Oxborough and Baker 2000; Baker et al. 2001; Lawson et al. 2008; Simkin et al. 2017b).

### Gas exchange measurements

The response of net photosynthesis (*A*) to intracellular CO_2_ (*C*_i_) was measured using a portable gas exchange system (CIRAS-1, PP Systems Ltd., Ayrshire, UK) as previously described (Simkin et al. 2017a and 2017b). Leaves were illuminated with an integral red–blue LED light source (PP systems Ltd., Ayrshire, UK) attached to the gas exchange system, and light levels were maintained at saturating PPFD of 1,000 *μ*mol m^−2^ s^−1^ for the duration of the *A*/*C*_i_ response curve. Measurements of *A* were made at ambient CO_2_ concentration (Ca) at 400 *µ*mol mol^−1^, before Ca was decreased to 550, 350, 215, and 60 *µ*mol mol^−1^ before returning to the initial value and increased to 740, 900, 1,140, 1,340, and 1,640 *µ*mol mol^−1^. Measurements were recorded after *A* reached a new steady state (1 to 2 min). Leaf temperature and vapor pressure deficit (VPD) were maintained at 25 °C and 1 ± 0.2 kPa, respectively. The maximum rates of Rubisco (*V*c_max_) and the maximum rate of electron transport for RuBP regeneration (*J*_max_) were determined and standardized to a leaf temperature of 25 °C based on equations from von Caemmerer and Farquhar (1981), Bernacchi et al. (2001), and Sharkey (2016). All points below 200 ppm were assigned as Rubisco limited and points above 300 ppm as RuBP regeneration limited as described (Sharkey 2016).

### Statistical analysis

All statistical analyses were done by comparing ANOVA, using Sys-stat (University of Essex, UK). The differences between means were tested using the post hoc Tukey’s test (SPSS, Chicago).

### Accession numbers

Sequence data from this article can be found in the GenBank/EMBL data libraries under accession numbers At3g26650 (NM113576) and At1g42970 (AY039961).

## Supplementary Material

kiad256_Supplementary_Data

## Supplemental data

The following materials are available in the online version of this article.

**Supplemental Figure S1.** Schematic representation constructs used for floral dipping.

**Supplemental Figure S2.** Original photos for Fig. 4.

**Supplemental Figure S3.** Growth analysis of nontransformed (WT) and azygous (Azy) experimental lines grown in low light.
