# Supplementary material for: Glyceraldehyde-3-phosphate dehydrogenase subunits A and B are essential to maintain photosynthetic efficiency
Source: Plant Physiol. 2023 Apr 26;192(4):2989–3000. doi: 10.1093/plphys/kiad256 (PMC11025378; doi:10.1093/plphys/kiad256)
Supplement: kiad256_Supplementary_Data [file kiad256_supplementary_data.pdf]

Figure S1

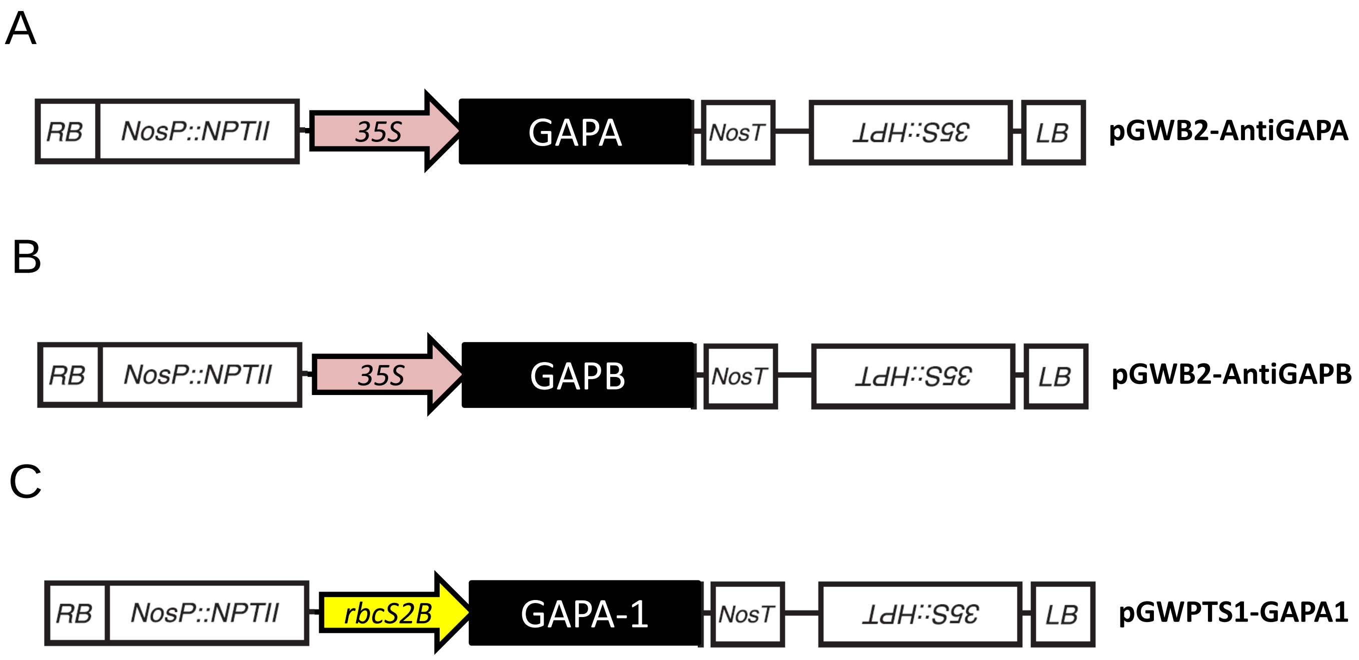

Supplemental Figure S1. Schematic representation of constructs used for floral dipping. (A) pGWB2-AntiGAPB antisense and (B) pGWPTS1-GAPA-1 antisense constructs and (C) pGWPTS1-AntiGAPA over-expression construct used to transform Arabidopsis (Col-0). cDNAs are under transcriptional control of the rbcS2B or 35s tobacco mosaic virus promoter (35S) followed by the nos 3' terminator (NosT). Constructs contained two selection cassettes, NosP::NPTII (Nopaline synthase promoter (NosP) and kanamycin resistance gene (NPTII)) and 35S::HPT (35S promoter (35S) and hygromycin resistance gene (HPT)). Following floral dipping, transgenic Arabidopsis plants were selected on both kanamycin and hygromycin containing medium (Nakagawa et al. 2007). RB; T-DNA right border, LB; T-DNA left border.

Figure S2

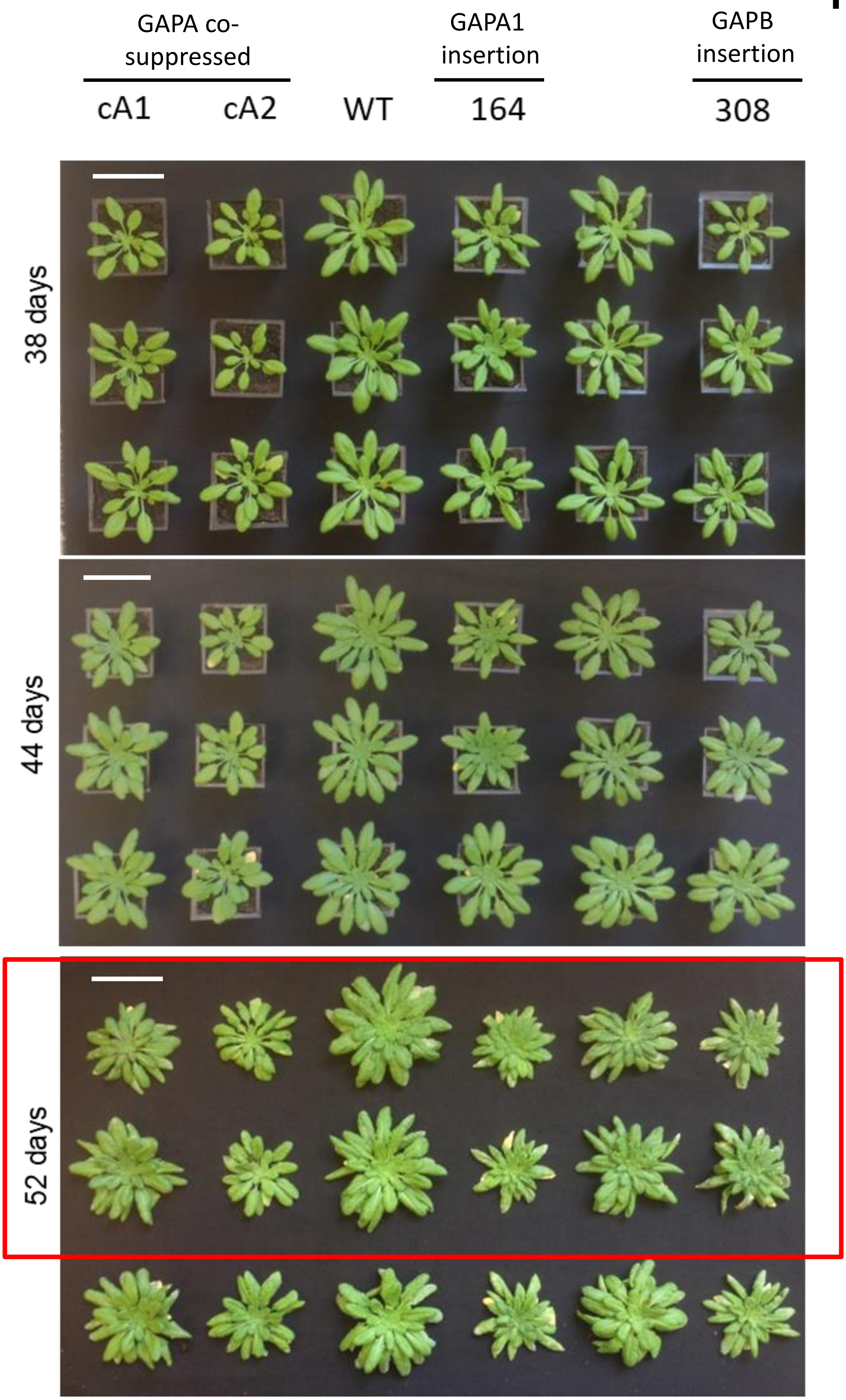

Supplemental Figure S2. Original photo for Figure 4. Growth analysis of control and experimental lines grown in low light. Plants were grown at 130 µmol m^{-2} s^{-1} light intensity in short days (8h/16h days) for 52 days. Lines co-suppressing GAPA (cA), GAPA1 insertion mutant (164) and GAPB insertion mutant (308) are represented. White bar represents a size of 6cm.

Figure S3

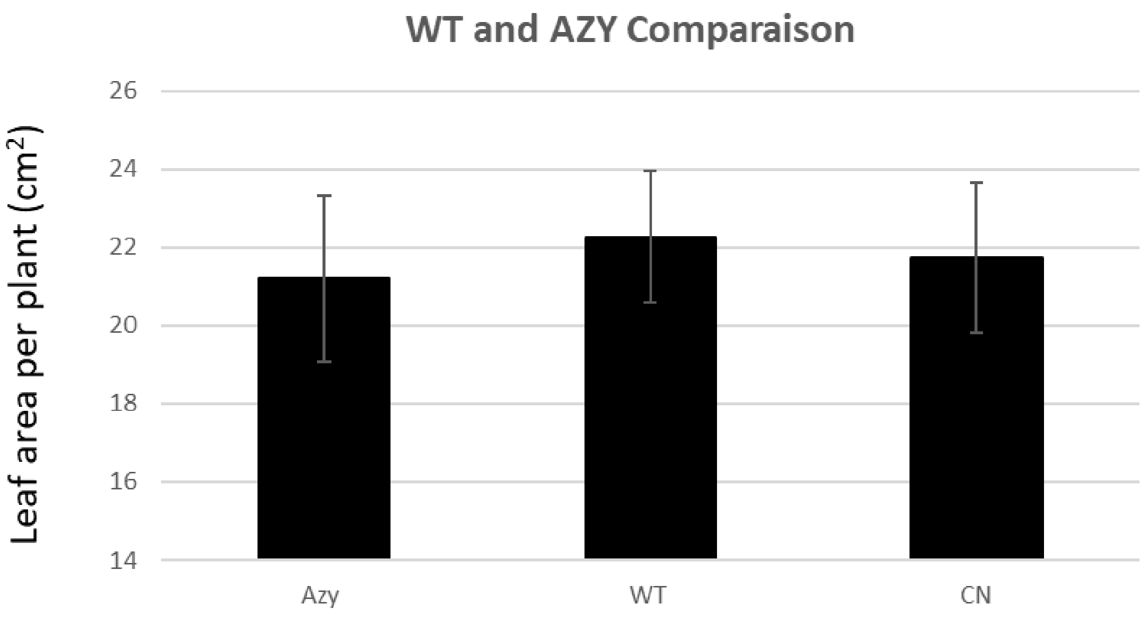

Supplemental Figure S3. Growth analysis of non-transformed (WT) and Azygous (Azy) experimental lines grown in low light. Plants were grown at 130 µmol m^{-2} s^{-1} light intensity in short days (8h/16h days) for 52 days. Azy plants were selected from segregating transgenic lines, verified by PCR for the absence of the transgene and seeds were collected and pooled. Azy seeds were grown alongside WT and homozygous transgenic lines in all experiments. No significant differences in leaf area are observed between Azy and WT. Standard deviations are shown. For clarity, in this manuscript, WT and Azy plants were combined into a single control group (CN).
